# An international survey of undergraduate trainees’ interests and expected teaching strategies in geriatric oncology

**DOI:** 10.1186/s12909-026-09426-x

**Published:** 2026-05-13

**Authors:** Ruth Zickermann, Mexhid Ferati, Ningbo Fan, Evelyn Plamper, Rabi Datta, ZiCheng Lyu, Nellie Horstmann, Judith Lohmann, Lena Pickert, Kah Poh Loh, Nicolò Matteo Luca Battisti, HuiXia Shen, Valentin Göde, David Iyú, Christiane Bruns, M. Cristina Polidori, Yue Zhao

**Affiliations:** 1 Department of General-, Visceral-, Thoracic- and Transplantation Surgery, University Hospital of Cologne, Cologne, Germany; 2 Informatics Department, Linnaeus University, Småland, Sweden; 3 Corporate Development, University Hospital of Cologne, Cologne, Germany; 4 Medical Faculty, University of Cologne, Cologne, Germany; 5 Klinik für Kinderchirurgie und Kinderurologie, Kinderkrankenhaus Amsterdamer Straße, Kliniken Köln, Cologne, Germany; 6 Ageing Clinical Research, Department II of Internal Medicine and Centre for Molecular Medicine Cologne, Faculty of Medicine, University of Cologne, University Hospital Cologne, Cologne, Germany; 7 Division of Hematology/Oncology, Department of Medicine James P. Wilmot Cancer Institute, Geriatric Hematology and Mobile Health Research, University of Rochester Medical Center, Rochester, NY USA; 8 Department of Medicine, Breast Unit, The Royal Marsden Hospital NHS Foundation Trust, London, UK; 9 Tongji University School of Medicine, International Education & Cooperation Office, Shanghai, PR China; 10 Department of Oncogeriatrics, Center of Geriatric Medicine, St. Marien- Hospital, Cologne, Germany; 11 Department of Physiology, University of Murcia, Murcia, Spain; 12 Instituto Murciano de Investigación Biosanitaria (IMIB)-Arrixaca, Unidad de Trasplante Hematopoyético y Terapia Celular, Murcia, Spain; 13 Faculty of Medicine, CECAD, University of Cologne, University Hospital Cologne, Cologne, Germany

**Keywords:** Geriatric oncology, Education, Geriatric assessment, Virtual reality, Augmented reality

## Abstract

**Background:**

Age is a critical driver of the worldwide increasing cancer incidence rates, and with the growing number of older adults diagnosed with cancer, there is a rising need for specialized care. However, geriatric oncology (GO) education remains underrepresented in medical curricula. The gap between demand and adequately trained providers requires the development of more robust GO training programs. This study examined the current state of GO education, and the areas of interest within GO and the expected teaching methods for this specialized content.

**Methods:**

A multi-center international online survey aimed at assessing the awareness and perceived significance of GO among early-career medical students and nursing trainees was conducted.

**Results:**

Among 158 participants, only 36.5% had oncology training and 19.6% geriatrics training. Despite this, GO was consistently rated as highly important, with no difference by prior GO training (*p* = 0.853). Participants expressed strongest interest in mental health, palliative care, and quality-of-care topics, while satisfaction with current training was moderate. Teaching was predominantly lecture-based, although more interactive approaches such as seminars and bedside teaching were preferred. Over half of participants were willing to contribute to improving GO education through feedback or volunteering.

**Conclusion:**

GO remains underrepresented in current training despite its high perceived importance among trainees. Participants preferred interactive teaching formats, particularly seminars and bedside teaching, and showed strong interest in mental health and palliative care topics. These findings support integrating geriatric oncology into curricula using more practice-oriented teaching strategies.

**Supplementary Information:**

The online version contains supplementary material available at 10.1186/s12909-026-09426-x.

## Introduction

Older adults with cancer are a growing, heterogeneous and particularly vulnerable patient population, presenting a unique set of challenges to healthcare professionals that requires specialized approaches. Due to the demographic shift towards an ageing population, 24 million new cancer cases are expected worldwide by 2035 among older adults, an increase of approximately 170% compared to 14 million cases in 2012. Older patients with cancer are estimated to account for more than 50% of the global cancer incidence burden in 2035 [1]. Given the increasing number of older adults with cancer, there is a pressing need for greater expertise and a more highly trained medical workforce to deliver optimal care. Therefore, expanding and enhancing geriatric oncology (GO) education within medical training is crucial for addressing this growing demand.

Older adults with cancer present a unique challenge to health care professionals due to their higher likelihood of frailty and multiple comorbidities that complicate care. Oncogeriatric health conditions include cognitive impairment, declined physiological functions, reduced organ, especially renal, function which can increase the risk of complications from locoregional and systemic anticancer treatments [2–4]. Due to their various health conditions older adults with cancer are often subject to polypharmacy which is associated with an increased risk of unwanted drug interactions, toxicities, and poor compliance [5]. As a result, older adults with cancer are frequently excluded from clinical trials, limiting the external validity of the evidence to guide treatment decisions and recommendations [6].

To overcome the barriers in oncogeriatric care, the cancer workforce requires upskilling to enable sophisticated and effective treatment decisions and medical care for older adults with cancer [7]. However, training in GO is often underrepresented in medical teaching and curricula [8, 9]. In the US, GO was reported to be the least oncology-related discipline that was included into the oncology fellowship curriculum with merely 32% of the teaching programs including GO formally into their oncology curriculum [10]. Consistent with this, a 2017 survey sent to hematology-oncology fellows in the US reported that over 50% had not participated in GO lectures and had overall low knowledge in clinical practice and experience with older adults with cancer. Similarly, a lack of structured education in geriatric oncology has also been reported in Europe and Japan [11, 12]. A Europe-wide COST Action 21,122 (PROGRAMMING) initiative has been launched to explore educational needs in geriatric medicine, but no results have been published [13].

To improve the care for older adults with cancer, stakeholders and caregivers have implemented different approaches. Institutions like International Society of Geriatric Oncology (SIOG) have put forward different initiatives to improve GO education, including webinars, online workshops and conferences [14, 15]. In addition, the Cancer and Aging Research Group (CARG) aims to bring together oncogeriatric researchers across the nation in a collaborative effort of designing and implementing clinical trials to improve the care of older adults with cancer. ASCO and ESMO have made available recommendations for a global GO curriculum [16]. However, implementation of GO training still faces many obstacles, including limited resources or access to GO training [14]. More recently, EUniWell ‘onco-aging’ working group was initiated in 2020 with seed funding. The initiative aims to develop a pilot program addressing key challenges in cancer care for older adults and to increase awareness among students in healthcare-related fields. The target group includes students from medicine, nursing science, life sciences, social work, and related disciplines.

This study aimed to explore the awareness and knowledge of GO and its role in medical education, particularly among medical students and nursing trainees. It also assessed current teaching strategies in GO and examined trainees’ preferences to inform the development of more effective educational approaches.

## Methods

### Study design and ethical approval

The study was conducted in accordance with the Declaration of Helsinki and was approved by the ethic committee of Medical Faculty of University of Cologne (ID: 21-1169). This study was designed as an international cross-sectional online survey. The survey was designed at the end of 2021, prior to the publication of the Consensus-Based Checklist for Reporting of Survey Studies [17], and therefore was not developed in accordance with this guideline.

### Survey

The survey [18] consisted of 12 main questions that branched into a total of 31 items assessing participants’ experiences and attitudes toward GO courses. The questionnaire was developed through an iterative process by a multidisciplinary working group with expertise in oncology, geriatrics, and medical education. Item generation was informed by a review of the relevant literature and the study objectives, aiming to capture key domains related to participants’ experiences and perceptions of GO education.

The preliminary version of the questionnaire was reviewed by subject-matter experts to evaluate content relevance, clarity, and comprehensiveness (content and face validity), and was subsequently refined based on their feedback. In addition, the survey was pilot-tested with a small group of medical students to assess readability, comprehension, and feasibility, leading to minor adjustments in wording and structure. The final survey included multiple-choice questions, yes/no questions, and 5-point Likert scale items to assess the frequency or intensity of participants’ experiences and attitudes. Several open-ended questions were also included to allow respondents to provide additional comments. The demographic section collected basic information, including age, gender, year of study, and institution. Prior to participation, all respondents reviewed an informed consent document outlining the study purpose, voluntary participation, confidentiality, and the right to withdraw at any time without penalty.

### Participants

Participants were informed about the nature and purpose of the survey, and responses were collected anonymously in 2022. Eligible participants included undergraduate medical students, nursing trainees, and other students in the early stages of healthcare-related education. The primary target group consisted of medical students and nursing trainees.

The survey was distributed via email, social media, and web-based channels, with support from the Linnaeus University Informatics Department. Participants could access the survey through a link (https://survey.lnu.se/Survey/29809) or QR code, which were widely shared. As the survey link could be freely distributed, a small number of participants from related disciplines beyond the primary target group may have been included. To promote transparency, participants could optionally provide their email address to receive the published article. A full list of questions is provided in the Supplementary material.

### Statistical analysis

Statistical analyses were performed using R software (version 4.4.1; R Foundation for Statistical Computing, Vienna, Austria). Group comparisons between participants with and without prior oncology training were conducted using the Mann–Whitney U test. A two-sided p-value < 0.05 was considered statistically significant. Data visualization was performed using the ggplot2 package.

## Results

### Demographics of the participants

A total of 158 participants responded to the multi-center online survey, which was accessible via a QR code and a web link. Due to the nature of the survey distribution, it was not possible to retrieve a response rate. Participants were predominantly aged between 18 and 25 (102; 64.6%) or between 25 and 35 (33; 21.0%) and female (102; 64.6%). Most participants were medical students (88; 55.7%) or enrolled in nursing training programs (49; 31.0%), mostly from Cologne, Murcia, or Tongji University (Table 1).

**Table 1:** Demographics of the participants

Demographics		*N*	%
Age	< 18	7	4.4%
	18–25	102	64.6%
	26–35	33	20.9%
	> 35	16	10.1%
Gender	Male	52	32.9%
	Female	102	64.6%
	Prefer not to provide	4	2.5%
	Other	0	0
Educational Program	Medicine	88	55.7%
	Nurse	49	31.0%
	Public Health/Epidemiology	3	1.9%
	Neuroscience	2	1.3%
	Psychology	0	0
	Veterinary	0	0
	Informatics	0	0
	Economics	0	0
	Other	16	10.1%
Country	Germany	72	45.6%
	Spain	46	29.1%
	China	22	13.9%
	USA	3	1.9%
	Other	15	9.5%

### Lack of GO training into medical and nursing education curricula

**Fig. 1:**
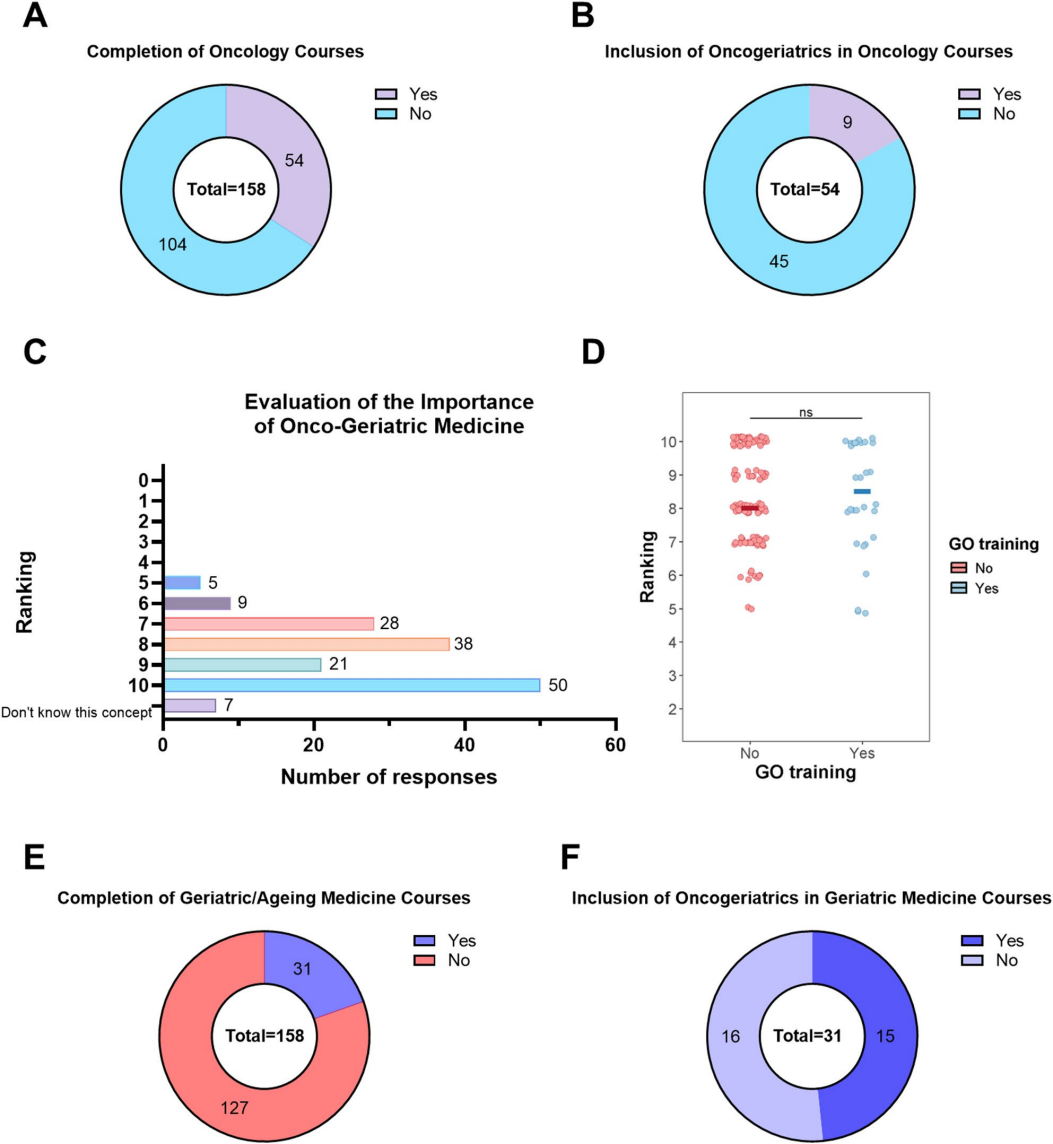
Education and perceived importance of geriatric oncology (GO) training among participants. Education and perceived importance of geriatric oncology (GO) training among participants. **A** Participation in oncology-related courses. **B** Reported inclusion of geriatric oncology content within completed oncology courses. **C** Participant ratings of the importance of geriatric oncology on a 0-10 scale. **D** Participant ratings of the importance of geriatric oncology on a 0-10 scale, stratified by prior GO training, each point represents an individual response and horizontal bars indicate median values. **E** Participation in formal geriatrics training (blue color scheme). **F** Reported inclusion of geriatric oncology content within geriatrics training programs

54 (36.5%) of all 158 participants had taken courses in oncology during their education. Of these 54 participants, 9 (16.7%) reported the inclusion of GO into their oncology courses (Fig. 1A-C). For the analysis of perceived importance, seven responses indicating “Don’t know the concept” were excluded. No significant difference was observed between participants with and without prior GO training (Mann–Whitney U test, *p* = 0.853; Fig. 1D). 31 participants (19.6%) had taken classes in geriatric medicine or medicine of ageing (Fig. 1E). Of these, 15 (48.4%) reported the inclusion of GO into their classes (Fig. 1F). Participants who reported to have participated in courses, including GO topics, reported that GO was allocated minor content in classes. The majority of participants who reported the inclusion of GO into their geriatric’s curricula estimated that less than 20% of the content was dedicated to GO. Less than 20% was the minimal option possible to select in the survey (Fig. S1A, B). All participants ranked the importance of GO inclusion into their curricula 5 or higher on a scale from 0 to 10, including 50 participants (31.7%) who ranked the importance 10 out of 10. Of the participants who already had classes in geriatrics, 27 (87.1%) confirmed the need for the inclusion of GO training into curricula (Fig. S1C). In the group of participants without previous training in geriatrics, 71 (55.9%) had an awareness of GO. 17 (10.8%) and 24 (15.2%) of all 158 participants were aware of SIOG and CARG, respectively.

### Area of focus for GO training

**Fig. 2:**
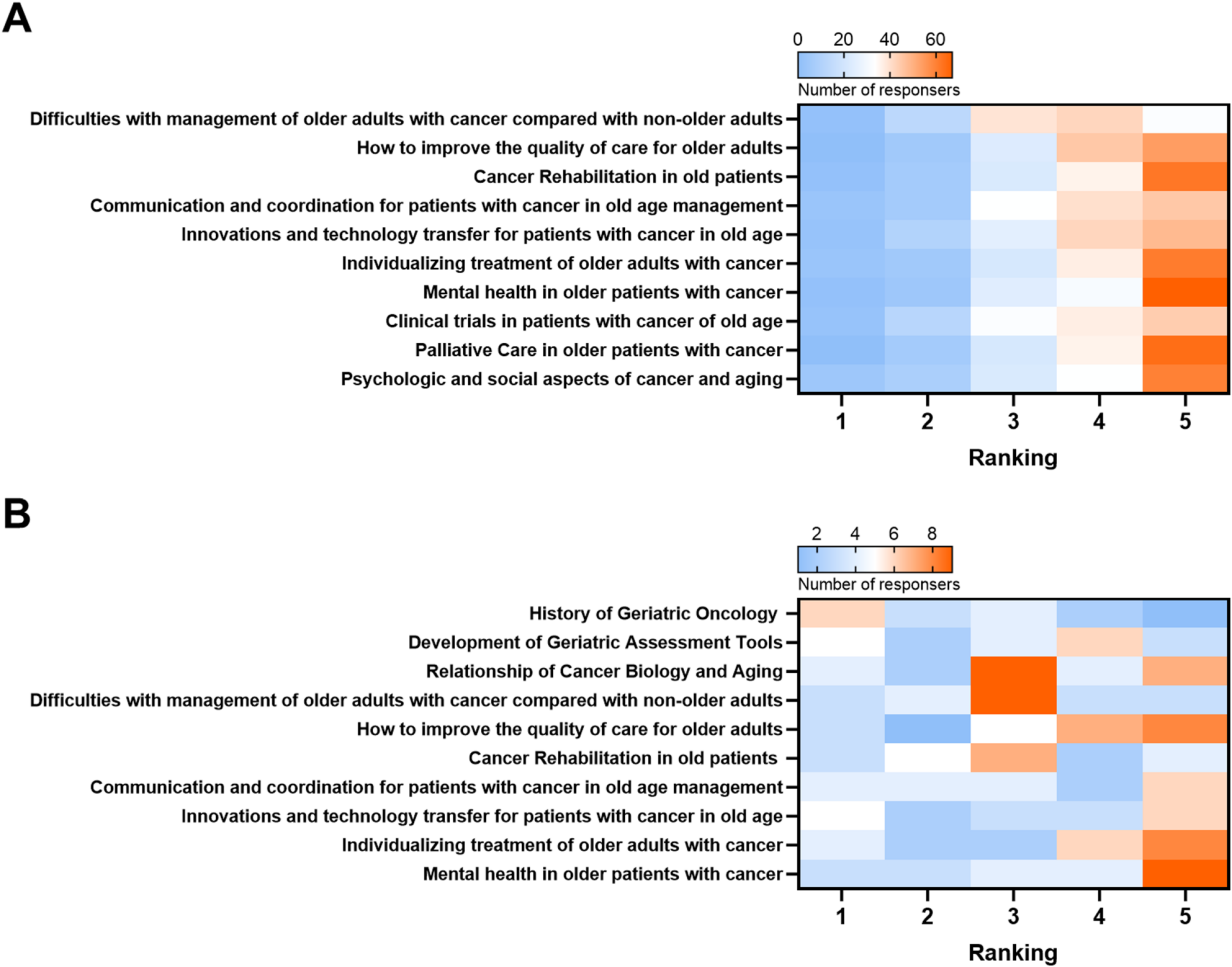
Participant interests in GO training topics **A** Interests of participants without prior GO training across GO-related topics. **B** Interests of participants with prior GO training across GO-related topics

Participants with training experience in geriatric medicine ranked their satisfaction with the current geriatric curriculum with a median score 3 on a scale from 0 to 5. With respect to geriatric assessment (GA), 25 participants (80.7%) with training in geriatrics confirmed the potential of GA to improve the quality of life (QoL) of geriatric patients and the majority, 20 participants (64.5%) considered GA an effective tool to support the guidance of a therapeutic strategy. Approximately half of the participants (51.6%, 16 participants) assigned GA a role in helping to detect frailty or dealing with toxicity in therapy decisions (Fig. S1D). With regard to learning objectives in GO training, participants with and without prior geriatrics training were especially interested in learning how to address mental health problems in older adults with cancer (Fig. 2). In accordance with that, participants indicated a learning interest in topics addressing hospice, palliative care or improving the quality of care (Fig. S2).

### Discrepancy between applied and expected teaching strategies

**Fig. 3:**
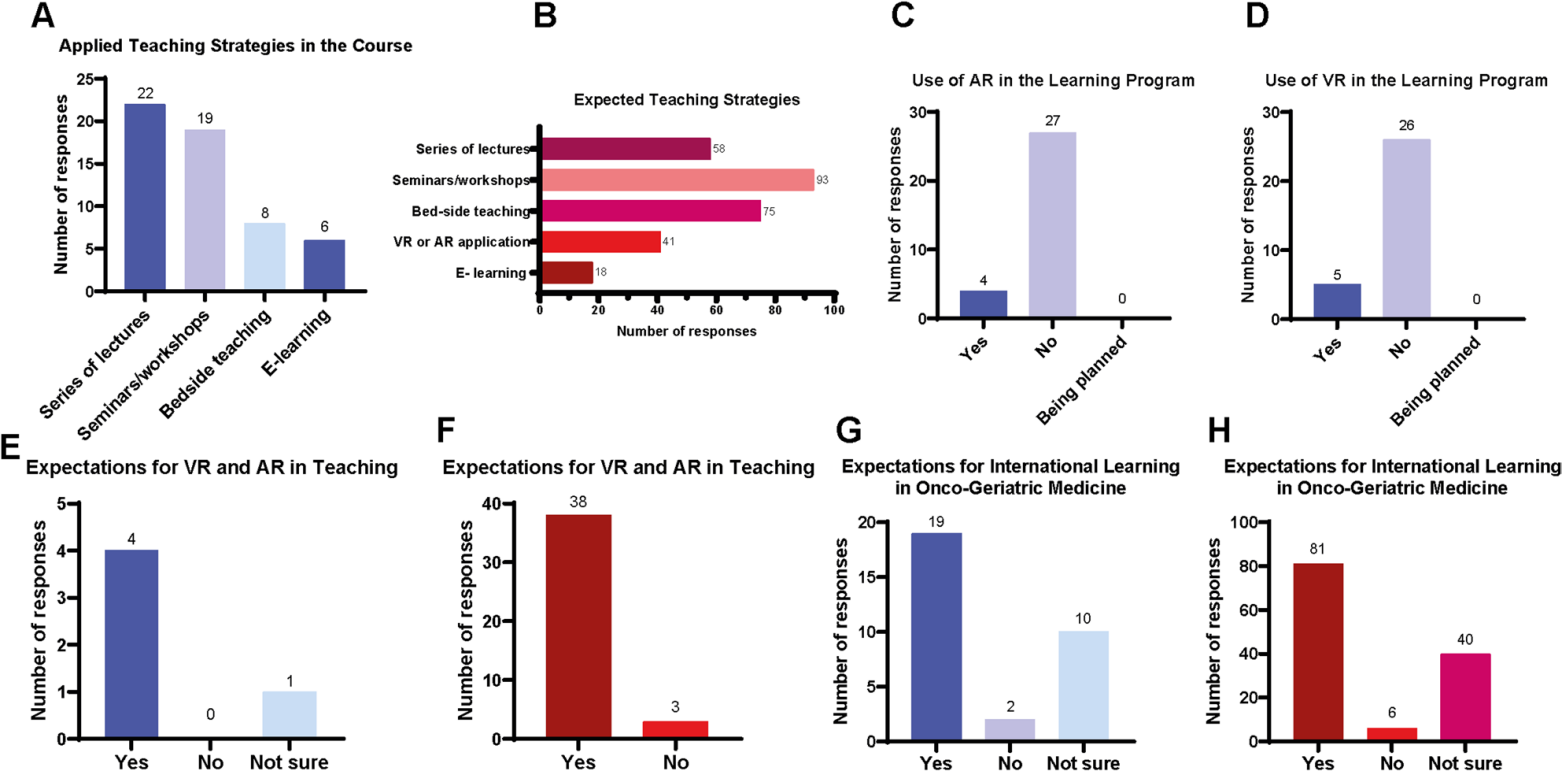
Teaching strategies in GO training. **A** Teaching strategies reported by participants with prior GO training. **B** Teaching strategies expected by participants without prior GO training. **C** and **D** Reported use of AR and VR in GO training. **E** Expected use of AR and VR among participants with prior AR or VR GO training. **F** Expected use of AR and VR among participants without prior AR or VR experience. **G** Expectations regarding international learning approaches among participants with prior GO training. **H** Expectations regarding international learning approaches among participants without prior GO training

The group with previous training in geriatrics (*n* = 31) reported that most teaching was conducted as a series of lectures or seminars and workshops. 8 (25.8%) participants reported training in bedside teaching and 6 (19.4%) participants in E-learning (Fig. 3A). Participants without previous geriatrics training (*n* = 127) most commonly expected seminars and workshops (93, 73.2%) and bedside teaching (75, 59.1%) as primary teaching strategies. Lectures were expected by 58 participants (45.7%), while fewer participants indicated VR/AR applications (41, 32.3%) and e-learning (18, 14.2%) (Fig. 3B).

Virtual Reality (VR) or Augmented Reality (AR) were only rarely applied. 5 (16.23%) and 4 participants (12.9%), respectively, reported the inclusion of VR or AR into teaching. Of these 5 participants with experience in AR or VR, 4 participants (80.0%) would expect more VR or AR in teaching. VR or AR application was expected from 41 participants (32.3%) and only 18 participants (14.2%) expected E-learning in GO teaching. Additionally, participants without previous experience in geriatric medicine (*n* = 127) reported that they would expect more international learning experiences (81; 63.8%). Yet, 40 participants (31.5%) also reported that they were unsure about expectations for international learning in GO (Fig. 3C-H).

### Willingness to participate in improving GO curricula

**Fig. 4:**
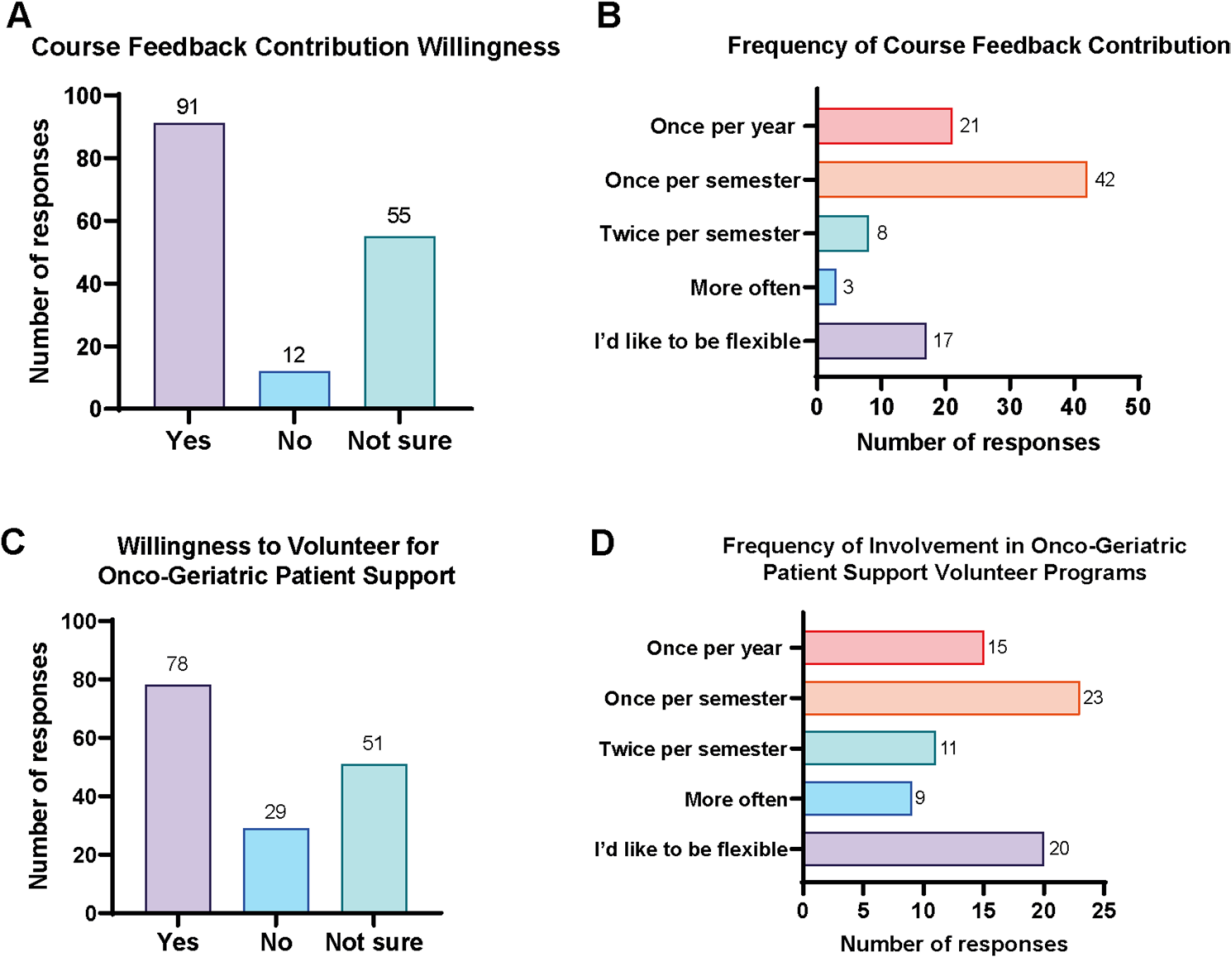
Willingness to support the improvement of GO curricula. **A** Participant responses regarding willingness to provide GO course feedback. **B** Preferred frequency of contribution to GO course feedback. **C** Participant responses regarding willingness to volunteer in oncogeriatric patient support. **D** Preferred frequency of contribution to oncogeriatric patient support among participants indicating willingness to contribute

In addition to reporting their preferences for learning objectives and teaching strategies in GO training, participants were also asked about their willingness to contribute to the improvement of GO courses through feedback or volunteering in patient support. The majority of participants (91; 57.6%) reported to be willing to give course feedback, mostly in a frequency of once a semester (42; 26.6%) or once a year (21; 13.3%), while 17 participants (10.8%) preferred a flexible schedule (Fig. 4A, B). Regarding patient support, 78 participants (49.4%) indicated willingness to volunteer, 29 (18.4%) were not willing, and 51 (32.4%) were unsure. Among those willing to help, most preferred to contribute once per semester (23; 29.5%), followed by 20 participants (25.6%) who preferred a flexible schedule (Fig. 4C, D). Overall, these findings indicate that many participants are willing to engage actively in supporting and improving GO training programs.

## Discussion

This multi-center, international online survey explored the awareness of GO among medical and nursing students and their expectations for GO training strategies as well as topics of particular interest. We found that GO is rarely included in medical curricula and, if taught, underrepresented in classes. Yet, the demand for GO training among medical students, with and without prior education in GO, is high. Furthermore, participants, having received training in GO, are confident that GO training can improve the care of older adults with cancer. Participants indicated that they were especially interested in learning about topics addressing mental health, QoL as well as hospice and palliative care with seminars and bedside teaching as most expected teaching strategies.

The global shortage of GO training has been described earlier, for example in a 2017 review about achievements and challenges of GO which acknowledged a ubiquitous incapacity to adequately manage the needs of older patients with complex chronic conditions [15]. For example, the review listed that the majority of geriatrics fellowship positions (56%) remained unfilled and only half of the geriatricians recertified in their subspecialty board in the US. While acknowledging that much has been done for improving the acceptance and increasing interest in geriatrics, e.g. by creating shorter fellowship programs and recognizing geriatrics as subspecialty, less than half of the countries reported the inclusion of geriatrics into medical school curricula. Furthermore, a qualitative study of hematology/oncology fellows noted that teachers may consider GO training unnecessary, as fellows frequently learn through daily experience with GO patients [19].

Our study contributes to existing literature by focusing on the needs of trainees in early stages of their education and by including their perspective on possible strategies to improve GO training. So far, most literature has evaluated GO training in fellowship programs, in particular in hematology and oncology fellowship programs [20]. Consistent with our results, Maggiore et al. found that hematology/oncology fellows rated GO training as “very important” on a Likert scale [19]. Yet only 19% of the fellows reported that their institution had a specific GO training program. In addition, Maggiore et al. showed that hematology/oncology fellows were lacking GO knowledge, for example regarding geriatric assessment (GA) or predictors of chemotherapy toxicity. Interestingly, there was no association between prior GO training and the frequency of correct answers in tests about GO knowledge which highlights the need to make GO training more valuable and effective [19]. To make GO training more accessible and successful, this study investigated topics of interest in GO training and expected teaching strategies of trainees. This can contribute to a re-evaluation of topics and applied teaching strategies in GO training.

The results of this survey indicate that participants were mostly interested in addressing mental health issues, palliative care and improving the QoL in older adults with cancer. While the recommended curriculum by ESMO/ASCO for oncology includes geriatrics and supportive and palliative care, it does not account for the relation of QoL and geriatrics in older adults with cancer [16]. Fellows in oncology who evaluated rotations in GO in interviews suggested that among others there was a need to address QoL and transitioning to palliative care discussions in teaching. Thus, psychological knowledge and skills were included into the GO curriculum based on the needs assessment [21].

Proficient teaching strategies are key to successful knowledge transmission. In order to overcome the challenge of a general lack of experts in GO, organizations such as ASCO and ESMO have developed e-modules [22]. While e-modules have the potential to make knowledge globally accessible to many trainees without the need of many experts generating content, accessibility can also be limited by costs of content creation and maintenance [22]. Offering GO e-learning to a price might reduce participation. For example, despite the increasing number of fellows enrolled in ASCO’s Education Essentials for Oncology Fellows (EEOF), participation in the general geriatric oncology module has remained consistently low, with only 2%, 12%, and < 1% of subscribed fellows accessing it between 2010 and 2013. As access to these modules requires an additional fee even for ASCO members, cost may represent a potential barrier limiting fellows’ engagement with this educational content [19].

In our study, we found that students reported limited use of E-learning and novel teaching strategies such as AR and VR. In addition, participants without prior geriatrics training ranked VR, AR and e-learning as least expected teaching strategies, and expected more workshops and seminars as well as bedside teaching. This finding primarily reflects participants’ expectations regarding the teaching formats they anticipate encountering in GO courses, rather than an explicit evaluation of preferred or most effective teaching strategies. These expectations may be influenced by the predominance of traditional lectures, seminars and bedside teaching in medical curricula, and should therefore be interpreted with caution. While this could suggest a perceived preference for teaching methods relying on personal interaction, it may also indicate limited exposure to digital and innovative formats such as e-learning, VR or AR. Similarly, hematology/oncology fellows have reported a desire for more clinical experience with older adults with cancer and more “hands-on” practice [19]. Notably, although based on a very small subgroup, participants with prior exposure to VR or AR reported a greater expectation for its use in GO teaching. This observation may reflect that familiarity with innovative methods could influence expectations, but is based on a very small subgroup and should not be considered generalizable.

This study indicates that participants were rather willing to contribute to GO course evaluation and volunteer for GO patients support. However, a substantial proportion of participants were unsure whether they would want to contribute to course feedback or GO patient support. Most participants were willing to offer their support once a semester, once a year or at a flexible rate. This uncertainty may partly reflect concerns about the time commitment required. Maggiore et al. documented that many fellows perceived time as a major barrier for doing GA with patients, but also for GO training. Fellows were also worried about time constraints regarding integration of more learning content and an extension of the curriculum in order to include GO as a specialty in their already existing curriculum [19]. Similarly, in our study, participants might have considered how their schedules and available time could affect their ability to provide course feedback or volunteer in GO patient support. Nevertheless, the overall willingness to engage suggests that trainees could represent a valuable resource for the ongoing development of GO education, for example through structured feedback mechanisms or optional involvement in educational or patient-support activities.

This study has several limitations related to the response rate, methodology, and the relatively small sample size, as well as the composition of the sample. The survey link and QR code were widely distributed by Linnaeus University through the EUniWell alliance and associated networks. As it was not possible to determine how often the link was accessed in total, a response rate could not be calculated. Although this limitation reduces the ability to fully assess the representativeness of the results, we nevertheless consider the findings valuable and worthy of dissemination to the wider research community. While some studies suggest that the relationship between response rates and nonresponse bias should be interpreted with caution, response rate reporting remains a standard methodological practice [23]. Consequently, we acknowledge that we are unable to assess potential bias arising from the fact that the survey captures responses only from individuals who chose to participate. Moreover, we cannot determine the proportion of such respondents within the broader population that received access to the survey.

Since the survey was conducted at the end of 2021, we did not follow the Consensus-Based Checklist for Reporting of Survey Studies [17] which may limit the standardization and comprehensive reporting of survey methodology and findings. Because the participant collective with prior education in geriatric medicine is small, the statistical significance is limited in questions regarding this collective. As participants were primarily trained in universities from Cologne (Germany), Shanghai (China) and Murcia (Spain) the informative value is limited to these training centers. While it can add new information to existing literature, the focus on trainees in the first years of their education might be questionable because GO goes hand in hand with a high degree of specialization which is usually part of the advanced educational pathway. In addition, the pre-selection of topics of interest and teaching formats could introduce a bias in the analysis. Nevertheless, we are still convinced that the outcomes of the study should be shared with the community and can motivate and guide further efforts to improve GO training.

## Conclusions

This survey provides valuable insights into the current state of GO education, highlighting that trainees perceive GO as highly important and express strong interest in topics such as mental health, palliative care, and quality of life. The findings underscore the need for further integration of GO content into curricula and suggest that more interactive, hands-on teaching approaches, such as seminars and bedside teaching, may better align with trainees’ expectations. While the study does not provide direct evidence for implementation, these insights may help inform the design and development of future GO curricula.

## Supplementary Information

Supplementary Material 1.

Supplementary Material 2.

## Data Availability

The data of this study are available under a transfer agreement from the corresponding author based on a reasonable request. There will be limitations on how data can be used or how long data will be available for.

## Abbreviations

GO: Geriatric oncology
QoL: Quality of Life
VR: Virtual reality
AR: Augmented reality
CARG: Cancer and Aging Research Group
SIOG: International Society of Geriatric Oncology
ASCO: American Society of Clinical Oncology
ESMO: European Society of Medical Oncology
GA: Geriatric assessment
