# Supplementary material for: An international survey of undergraduate trainees’ interests and expected teaching strategies in geriatric oncology
Source: BMC Med Educ. 2026 May 13;26:755. doi: 10.1186/s12909-026-09426-x (PMC13169813; doi:10.1186/s12909-026-09426-x)
Supplement: Supplementary file 1 — Supplementary Material 1. [file 12909_2026_9426_MOESM1_ESM.docx]

**Survey Questions**

| **Questions/Sub question** | **Response options** |
| --- | --- |
| **Q1.** Age | < 18,  18-25,  26-35,  >35 |
| **Q2.** Gender | Male,  Female,  I’d rather not say,  Other [please specify] |
| **Q3.** Medical School or other healthcare associated educational program (nurse, MPH etc): | Leiden University,  Linnaeus University,  Semmelweis University,  University of Birmingham,  University of Cologne,  University of Florence,  University of Nantes,  Other [please specify] |
| **Q4.** Education program | Veterinary  Medicine  Nurse  Public Health/Epidemiology  Neuroscience  Psychology  Informatics  Economics  Other [please specify] |
| **Q5.** Provide the year of your study (fill in a number) | [Open ended] |
| **Q6.** Have you already taken courses in oncology? | Yes,  No |
| **Q6.1.** Was the subject oncogeriatrics (or geriatric oncology) part of your oncology courses? | Yes,  No |
| **Q7.** How do you evaluate the importance of Onco-Geriatric Medicine? | Ranking 0-10 (10 is top improtance) |
| **Q8.** Have you already taken courses in Geriatric medicine / Ageing Medicine? | Yes,  No |
| **Q8Y1.** Was the subject oncogeriatrics (or geriatric oncology) part of your Geriatric medicine courses? | Yes,  No |
| **Q8Y2.** How much content is dedicated to Onco-Geriatric medicine in your Geriatric/Ageing Medicine course? | <20%,  20%-50%,  >50% |
| **Q8Y3.** How do you like the current Geriatric medicine / Ageing Medicine course/learning program? | 5-scale Likert Scale |
| **Q8Y4.** Do you think if there is a need for more information and training in Geriatric oncology? | Yes,  No,  Not Sure |
| **Q8Y5.** How do you think geriatric assessment could help the medical care in clinical practice? (multiple choice) | [Multiple choice]  Support for guidance/definition of therapeutic strategy;  Improve the quality of life;  Help to detect the fragility;  Predict the toxicity of the treatment to some extent  Reducing the risk of severe toxicity on cancer treatment  Other [please specify] |
| **Q8Y6.** Which teaching strategies were applied in the course (multiple selection is available) | [Multiple choice]  Series of lectures;  Seminar/Workshops;  Bed-side teaching;  E-learning via____platform |
| **Q8Y7.** Was VR (Virtual Reality) applied in the course/learning program? | Yes,  Not yet,  Being planned |
| **Q8Y8.** Was AR (Augmented Reality) applied in the course/learning program? | Yes,  Not yet,  Being planned |
| **Q8Y8.1.** Are you expecting more VR (Virtual Reality), or AR (Augmented Reality) applications in the teaching/learning activities? | Yes,  No,  Not Sure |
| **Q8Y9.**  Please rank the following topics based on how good they were at attracting your attention in yourcurrent course associated to Onco-Geriatric medicine. If the topic isunknown, leave it empty*. | Ranking 0-5 |
| **Q8Y10.** Additional questions you are interested to know about Onco-Geriatrics | [Open ended] |
| **Q8Y11.** Are you expecting more international learning experience of Onco-Geriatric medicine? | Yes,  No,  Not sure |
| **Q8N1.** Have you ever heard or known about Onco-Geriatrics before? | Yes,  No |
| **Q8N2.** Are the items/topics below of interest for your learning? (multiple selection is available) | [Multiple choice]  Geriatric assessment;  Reasons to perform a geriatric assessment;  The best timeframe to perform a geriatric assessment;  Current geriatric screening tools;  Status on geriatric assessment for radiation and surgical oncology;  Strategies on developing an Onco-geriatric unit;  Palliative care;  Hospice care;  Other [please specify] |
| **Q8N3.** Which teaching strategies would you expect for the course (multiple selection is available) | [Multiple choice]  Series of lectures;  Seminars/workshops;  Bed-side teaching;  VR (Virtual Reality) or AR (Augmented Reality) application in the course;  E-learning via _______ platform |
| **Q8N4.** Please rank the following items based on how interesting they wouldbe to learn more about**. | Ranking 0-5 |
| **Q8N5.** Are you expecting more international learning experience of Onco-Geriatric medicine? | Yes,  No,  Not sure |
| **Q9.** Are you willing to contribute to Onco-Geriatric medicine course feedback? | Yes,  No,  Not sure |
| **Q9.1.** How often? | Once per year,  Once per semester,  Twice per semester,  More often,  I’d like to be flexible |
| **Q10.** Are you willing to be involved in volunteer programs supporting cancer patients in old age? | Yes,  No,  Not sure |
| **Q10.1.** How often? | Once per year,  Once per semester,  Twice per semester,  More often,  I’d like to be flexible |
| **Q11**. Do you know the International Society of Geriatric Oncology (SIOG)? | Yes,  No |
| **Q12**. Do you know the Cancer and Aging Research Group (CARG)? | Yes,  No |
| **Q13**. Anything you want to add? | [Open ended] |

* topics in **Q8Y9**: History of Geriatric Oncology/Onco-Geriatric medicine; Development of Geriatric Assessment Tools in Oncology application; Relationship of Cancer Biology and Aging; Difficulties with management of older adults with cancer compared with non-older adults; How to improve the quality of care for older adults; Cancer Rehabilitation in old patients; Communication and coordination for patients with cancer in old age management; Innovations and technology transfer for patients with cancer in old age; Case discussion for patients with cancer in old age management/ individualizing treatment of older adults with cancer; Mental health in of older adults with cancer.

** items in **Q8N4**: Difficulties with management of older adults with cancer compared with non-older adults; How to improve the quality of care for older adults; Cancer Rehabilitation in old patients; Communication and coordination for patients with cancer in old age management; Innovations and technology transfer for patients with cancer in old age; Case discussion for patients with cancer in old age management/ individualizing treatment of older adults with cancer; Mental health in of older adults with cancer; Clinical trails in patients with cancer in old age; Palliative Care in older patients with cancer; Psychologic and social aspects of cancer and aging.
