# Supplementary material for: An international survey of undergraduate trainees’ interests and expected teaching strategies in geriatric oncology
Source: BMC Med Educ. 2026 May 13;26:755. doi: 10.1186/s12909-026-09426-x (PMC13169813; doi:10.1186/s12909-026-09426-x)
Supplement: Supplementary file 2 — Supplementary Material 2. [file 12909_2026_9426_MOESM2_ESM.docx]

**Figures**

**
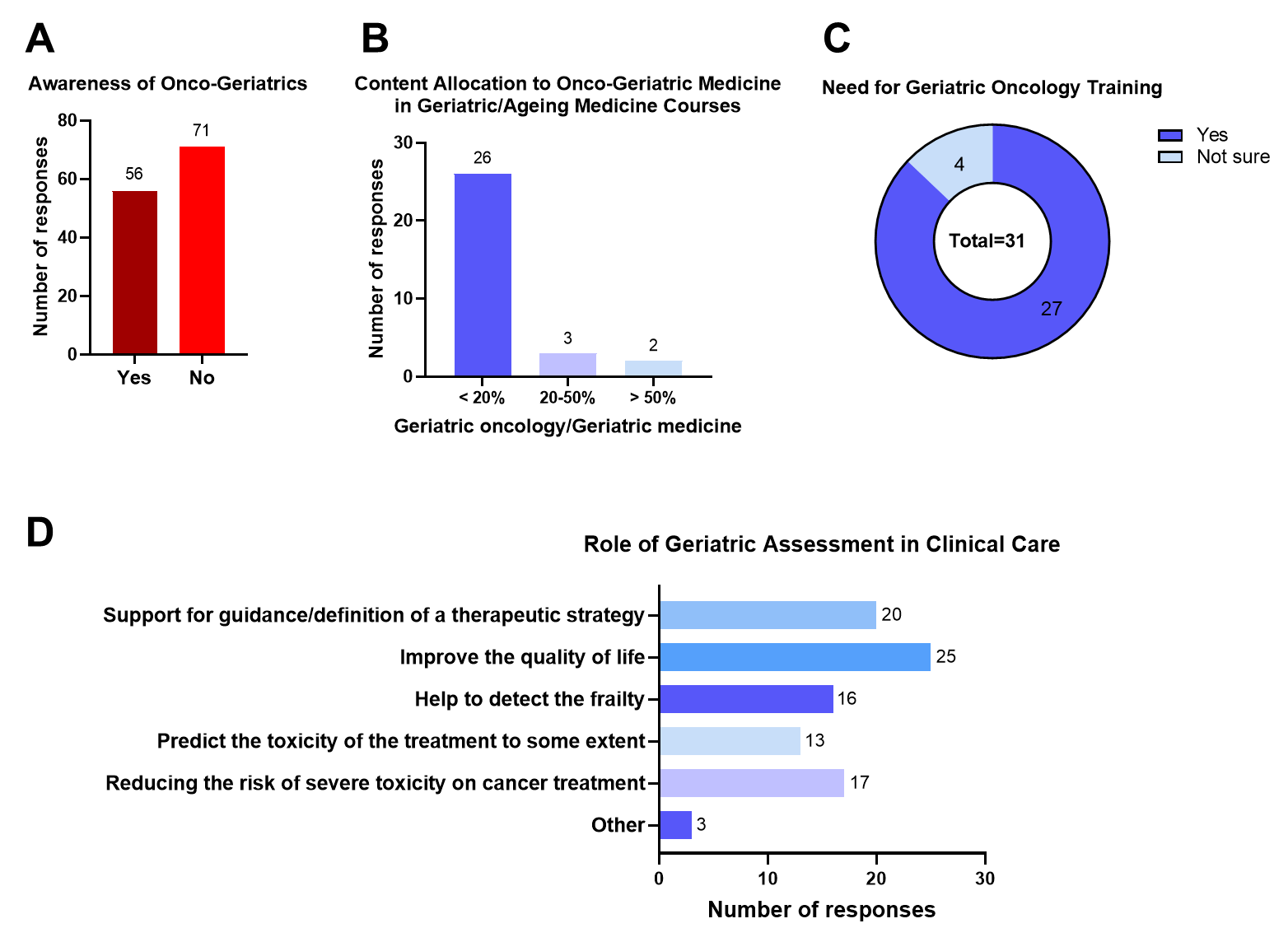
**

**Fig.S1** (A) Awareness of GO among participants without prior geriatrics education (red color scheme). (B) Proportion of GO course content allocated within existing teaching programs. (C) Participant responses regarding the perceived need for GO training. (D) Perceived roles of geriatric assessment in oncology care among participants with geriatrics training, including effects on QoL, therapeutic strategy, treatment-related toxicity, and detection of frailty.

**
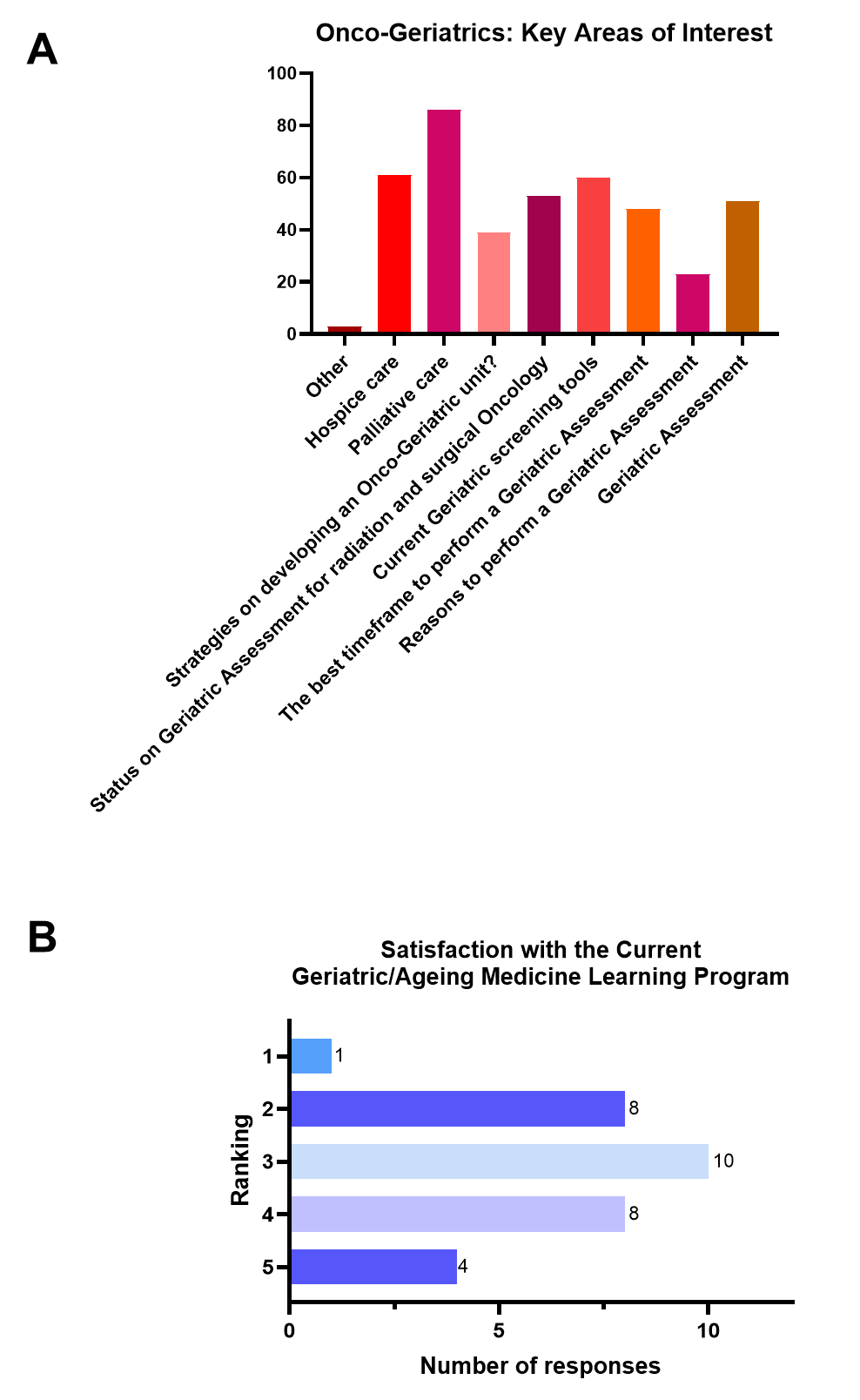
**

**Fig.S2** (A) Interests in GO-related topics among participants without prior geriatrics training. (B) Participant ratings of satisfaction with the current geriatrics curriculum among participants with prior geriatrics training.
